# Evaluating the Impact of Sequencing Depth on Transcriptome Profiling in Human Adipose

**DOI:** 10.1371/journal.pone.0066883

**Published:** 2013-06-24

**Authors:** Yichuan Liu, Jane F. Ferguson, Chenyi Xue, Ian M. Silverman, Brian Gregory, Muredach P. Reilly, Mingyao Li

**Affiliations:** 1 Department of Biostatistics and Epidemiology, Perelman School of Medicine at the University of Pennsylvania, Philadelphia, Pennsylvania, United States of America; 2 Cardiovascular Institute, Perelman School of Medicine at the University of Pennsylvania, Philadelphia, Pennsylvania, United States of America; 3 Department of Biology, Perelman School of Medicine and School of Arts and Science at the University of Pennsylvania, Philadelphia, Pennsylvania, United States of America; Auburn University, United States of America

## Abstract

Recent advances in RNA sequencing (RNA-Seq) have enabled the discovery of novel transcriptomic variations that are not possible with traditional microarray-based methods. Tissue and cell specific transcriptome changes during pathophysiological stress in disease cases versus controls and in response to therapies are of particular interest to investigators studying cardiometabolic diseases. Thus, knowledge on the relationships between sequencing depth and detection of transcriptomic variation is needed for designing RNA-Seq experiments and for interpreting results of analyses. Using deeply sequenced Illumina HiSeq 2000 101 bp paired-end RNA-Seq data derived from adipose of a healthy individual before and after systemic administration of endotoxin (LPS), we investigated the sequencing depths needed for studies of gene expression and alternative splicing (AS). In order to detect expressed genes and AS events, we found that ∼100 to 150 million (M) filtered reads were needed. However, the requirement on sequencing depth for the detection of LPS modulated differential expression (DE) and differential alternative splicing (DAS) was much higher. To detect 80% of events, ∼300 M filtered reads were needed for DE analysis whereas at least 400 M filtered reads were necessary for detecting DAS. Although the majority of expressed genes and AS events can be detected with modest sequencing depths (∼100 M filtered reads), the estimated gene expression levels and exon/intron inclusion levels were less accurate. We report the first study that evaluates the relationship between RNA-Seq depth and the ability to detect DE and DAS in human adipose. Our results suggest that a much higher sequencing depth is needed to reliably identify DAS events than for DE genes.

## Introduction

A transcriptome represents a collection of all transcribed sequences present in a given cell. Unlike a genome, which is roughly fixed, the composition of the transcriptome can be quickly restructured by changing the rate of synthesis or decay of individual mRNAs in response to external environmental conditions. The characterization of gene expression in cells has long been of interest to researchers because alterations in transcriptome profiles in response to specific biological stimuli provides valuable insights for interpreting functional elements of the genome and understanding disease pathogenesis. Tissue and cell specific transcriptomic changes during stress, in disease versus health and in response to therapies are of particular interest to investigators studying cardiometabolic diseases.

In the past decade, microarrays have been the method of choice for transcriptomics studies due to their ability to measure thousands of transcripts simultaneously [1]. However, microarrays are subject to biases in hybridization strength, and potential for cross-hybridization to probes with similar sequences [2]. Additionally, microarrays are unable to identify novel genes and splicing events because of their reliance on existing gene models. RNA sequencing (RNA-Seq) is an emerging approach for transcriptome profiling that allows an unbiased survey of the entire transcriptome in a high-throughput manner [3]. With deep coverage and single nucleotide resolution, RNA-Seq provides a platform to determine differential expression of genes or isoforms [4], alternative splicing (AS) [5], non-coding RNAs [6], post-transcriptional modifications [7], [8], and gene fusions [9].

Although RNA-Seq has revolutionized transcriptomics studies, the expense of sequencing is still a major limiting factor to obtaining highly informative datasets. Thus, knowledge of the relationships between sequencing depth and transcriptomic variation detection is critical for proper design of RNA-Seq experiments and for understanding the characteristics of the results. Sequencing depth represents the total number of sequenced reads, which can be increased by using more lanes. Despite its importance, empirical assessment of the impact of sequencing depth on transcriptome profiling is limited. A few studies have examined the relationship between sequencing depth and the detection of expressed genes and isoforms [10], [11], [12]. However, results from these studies cannot be extrapolated to questions that are beyond the detection of static gene or isoform expression. In many cardiovascular studies, it is often of interest to compare transcriptomic profiles between different physiological conditions, disease states and drug therapies. Therefore, it is imperative to investigate the effect of sequencing depth on detection of transcriptomic changes in order to properly design and interpret these types of experiments in disease relevant tissues.

By utilizing deeply sequenced RNA-Seq samples obtained from adipose of a single healthy individual before and after systemic administration of endotoxin (LPS), we set out to evaluate the effect that sequencing depth has on the statistical analysis of RNA-Seq data in an evoked model of innate immune stress of direct relevance to cardiometabolic disease [13], [14], [15], [16]. Specifically, we evaluated how sequencing depth relates to the identification of expressed genes and AS as well as to the detection of LPS-modulated differential gene expression and differential AS. Findings from our investigation provide a practical guide for researchers when designing RNA-Seq experiments in cells and tissues of direct relevance to cardiometabolic disease.

## Materials and Methods

### Study Subject

The subject is a healthy Caucasian female individual selected from the Genetics of Evoked responses to Niacin and Endotoxemia (GENE) study (n = 294), a recently completed National Institute of Health-sponsored experimental endotoxemia protocol performed at the University of Pennsylvania (UPenn) [17]. The GENE study was performed with the approval of UPenn’s Institutional Review Board after written informed consent was obtained from all research participants. All subjects underwent an inpatient endotoxemia protocol lasting approximately 40 hours, including a pre-LPS acclimatization phase, administration of IV LPS bolus (1 ng/Kg), and a 30-hour post-LPS phase. As described earlier [16], [18], samples of gluteal subcutaneous fat tissue were obtained at baseline, 4, 12 and 24 hours following LPS using a liposuction catheter under local anesthesia and snap-frozen for subsequent RNA extraction. Based on prior microarray mRNA profiling in independent samples [18], we selected baseline and 4-hr adipose samples for RNA-Seq.

### RNA-Seq Library Preparation and Sequencing

The RNA was extracted using RNeasy Lipid Tissue total RNA mini kit (Qiagen, Valencia, CA), underwent quality control using the Agilent Bioanalyzer (Agilent, Santa Clara, CA). Poly-A library preparation and sequencing were performed at the Penn Genome Frontiers Institute’s High-Throughput Sequencing Facility using Illumina’s HiSeq 2000 with four lanes per sample which generated 2×101 bp paired-end reads. Technical replicate data from the same individual were generated from independent library preparations and sequenced using two samples per lane.

Poly-A library preparation and sequencing were performed at the Penn Genome Frontiers Institute’s High-Throughput Sequencing Facility per standard protocols. Briefly, we generated first-strand cDNA using random hexamer-primed reverse transcription, followed by secondstrand cDNA synthesis using RNase H and DNA polymerase, and ligation of sequencing adapters using the TruSeq RNA Sample Preparation Kit (Illumina, San Diego, CA). Fragments of ∼350 bp were selected by gel electrophoresis, followed by 15 cycles of PCR amplification. The prepared libraries were then sequenced using Illumina’s HiSeq 2000 with four lanes per sample which generated 2×101 bp paired-end reads. Technical replicate RNA-Seq data from the same individual were generated from independent library preparations and sequenced using two samples per lane.

### Alignment of RNA-Seq Reads

The RNA-Seq data were aligned to the hg19 reference genome using Tophat v1.3.3 with default options [19]. In order to eliminate mapping errors and reduce potential mapping ambiguity due to homologous sequences, several filtering steps were applied. Specifically, we required the mapping quality score of each read to be ≥30, reads from the same pair were mapped to the same chromosome with expected orientations and the mapping distance between the read pair was <500,000 bp, and each read was uniquely mapped to the genome. All subsequent analyses were based on filtered alignment files.

### Random Sampling of Aligned RNA-Seq Reads

To investigate the effect of sequencing depth on analysis of RNA-Seq data, after removing reads mapped to the mitochondrial genome (based on Tophat alignment), we randomly selected reads from the filtered alignment files (482 million (M) reads for pre-LPS; 519 M reads for post-LPS) and created subsets with 5 M, 10 M, 15 M, 20 M, 25 M, 50 M, 75 M, 100 M, 150 M, 200 M, 300 M, 400 M reads for both the pre- and post-LPS samples.

For empirical validations, we used technical replicate RNA-Seq data generated from independent library preparations using two samples per lane which resulted in 67 M and 65 M reads for the pre- and post-LPS samples, respectively, with 36 M reads and 33 M reads after filtering and removal of reads mapping to the mitochondrial genome.

### Analysis of Gene Expression

Transcripts were assembled and gene expression levels were estimated using Cufflinks v1.3.0 [4]. A gene was declared as expressed if the FPKM (Fragments Per Kilobase of exon per Million fragments mapped) value was >0. For each gene, we compared the gene expression levels between pre- and post-LPS administration using the cuffdiff option in Cufflinks. A gene was declared as differentially expressed if the FDR adjusted p-value was ≤0.05.

### Analysis of Alternative Splicing

To identify alternative splicing (AS) events, we used MATS [20], a computational tool that detects differential AS events from RNA-Seq data. We favored MATS over other tools (e.g., MISO [21] and DiffSplice [22]) because of our experimental design and the ease of use and robustness of the program. MATS tests that the difference in the exon or intron inclusion level of a gene (defined by refSeq in our analysis) between two conditions exceeds a user-defined threshold (0.05 in our analysis). From RNA-Seq data, MATS can automatically detect AS events corresponding to all major types of AS, including exon skipping, mutually exclusive exons, alternative 5′ splice site, alternative 3′ splice site, and intron retention. An AS event was declared if the inclusion level of an exon or an intron was between 0 and 1. A differential AS event was declared if the FDR adjusted p-value was ≤0.05.

## Results

### Clinical Characteristics of the Study Subject

Clinical and biochemical responses to LPS in all European Ancestry participants in the GENE study as well as for the study subject are shown in **Table S1**. Compared to GENE participants, the selected study subject had similar baseline characteristics with normal blood pressure, blood glucose, and plasma lipoproteins. We selected this subject due to their high biochemical inflammatory response (84 percentile peak IL-6, 64 percentile peak TNFα, and 87 percentile peak temperature in the 24 hours post-LPS) (**Table S1**), supporting a robust modulation of the transcriptome.

### RNA-Seq Data Alignment

We obtained 912 million (M) and 1,040 M reads for the pre- and post-LPS samples, respectively, with a high mapping rate, 85% and 82% of the reads mapped to the reference genome for the pre- and post-LPS samples, respectively, and 72% and 69% of the reads uniquely mapped and properly filtered (**Table 1**). In our analysis, we only considered reads from autosomal and sex chromosomes, and this left 482 M filtered reads pre-LPS and 519 M filtered reads post-LPS. For ease of notation, we denote the 482 M and 519 M filtered datasets both as 500 M, and assume results from the analyses of these two datasets provide a comprehensive catalogue of transcriptomic variation.

**Table 1:** Mapping statistics.

Sample	Time	Reads	Reads mapped (%)	Reads after filtering (%)	Autosomal and sex chromosome reads after filtering (%)
Original	Pre-LPS	911,584,508	771,290,702 (85%)	655,529,906 (72%)	481,769,060 (53%)
	Post-LPS	1,039,937,222	856,379,122 (82%)	718,792,994 (69%)	518,576,050 (50%)
Technical replicate	Pre-LPS	66,603,980	57,113,510 (86%)	49,217,950 (74%)	36,253,892 (54%)
	Post-LPS	64,824,708	53,726,630 (83%)	45,005,478 (69%)	32,587,354 (50%)

Data were aligned to the hg19 reference genome using Tophat v1.3.3.

### Impact of Sequencing Depth on Analysis of Gene Expression

#### Detection of gene expression

**Figure 1:**
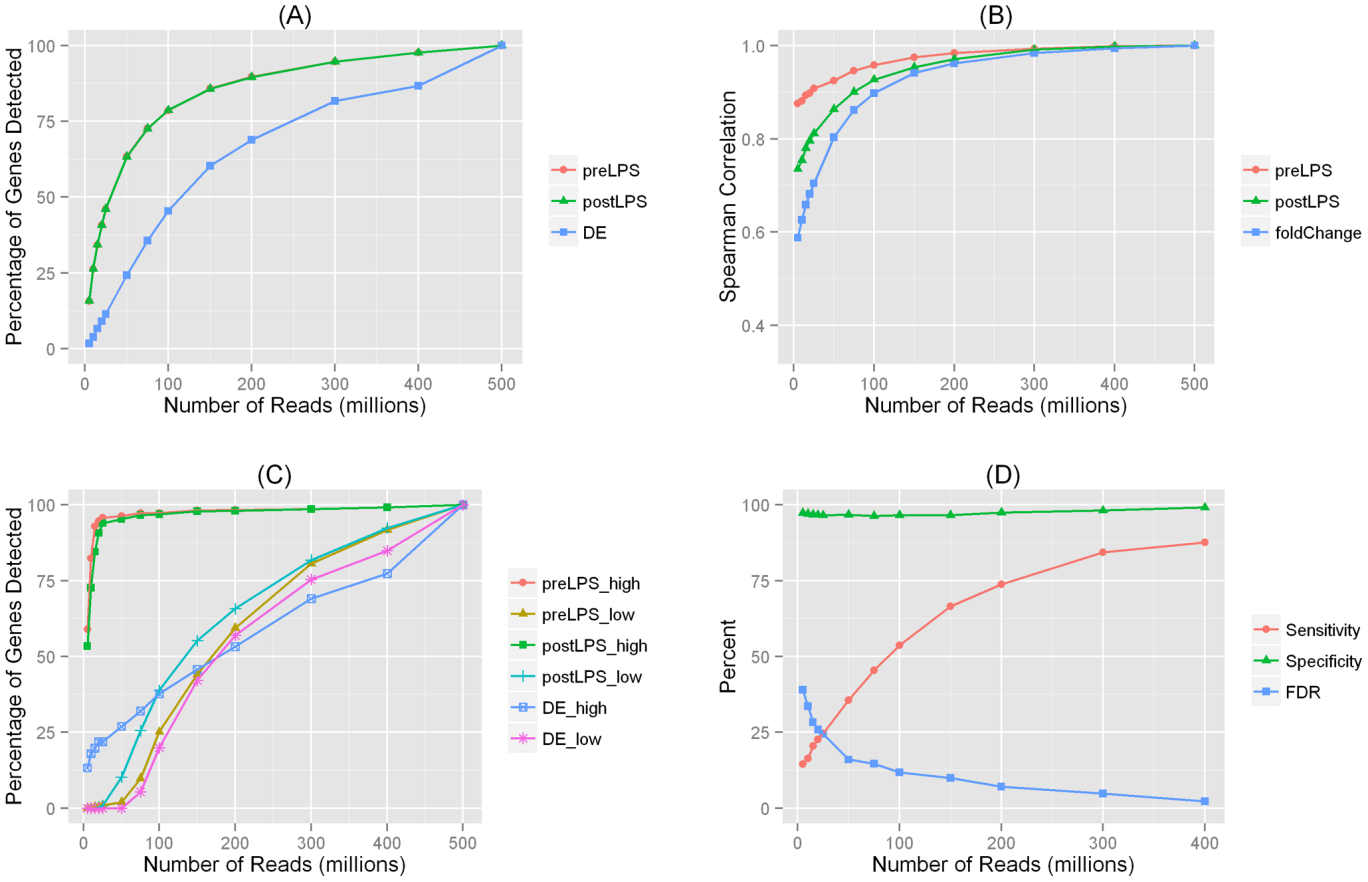
Analysis results for differentially expressed (DE) genes in adipose. (**A**) **Percentage of detected expressed genes and differentially expressed (DE) genes for datasets with various sequencing depths.** PreLPS: detection rate for expressed genes in the pre-LPS sample; post-PLS: detection rate for expressed genes in the post-LPS sample; DE: detection rate for DE genes. The curves for pre-LPS and post-LPS samples overlap, although the numbers of detected genes were different (Table S1). (**B**) **Spearman correlation between FPKM values in datasets with various sequencing depths and FPKM values in the 500 M-read datasets.** PreLPS: correlation of FPKM values in the pre-LPS sample; postLPS: correlation of FPKM values in the post-LPS sample; fold-change: correlation of the fold change of FPKM values. (**C**) **Percentage of detected DE genes according to gene expression levels.** PreLPS_high: detection rate for gene expression in highly expressed genes in the pre-LPS sample; preLPS_low: detection rate for gene expression in lowly expressed genes in the pre-LPS sample; postLPS_high: detection rate for gene expression in highly expressed genes in the post-LPS sample; postLPS_low: detection rate for gene expression in lowly expressed genes in the post-LPS sample; DE_high: detection rate for DE genes in highly expressed genes; DE_low: detection rate for DE genes in lowly expressed genes. (**D**) **Performance of DE genes detected in datasets with various sequencing depths.**

To assess the relationship between sequencing depth and gene expression, we divided our 500 M datasets into smaller subsets randomly and analyzed how the detection of a gene varies with sequencing depth. For the pre-LPS sample, we found that with 5 M reads, only 16% of the expressed genes were detected. The detection rate quickly increased to 79% when the sequencing depth increased to 100 M (**Figure 1(A)** **; Table S2**). After the sequencing depth reached 150 M, the percentage of additionally detected genes became less pronounced and each additional 100 M reads only offered 3% to 5% more genes, suggesting that the improvement of sequencing depth after 150 M had relatively less impact on detecting low abundance genes. We observed an almost identical pattern for the detection rate for the post-LPS sample, although the numbers of detected genes were different from the pre-LPS sample (**Table S2**).

#### Detection of differential gene expression

Because detection of differentially expressed (DE) genes is a key focus for many cardiometabolic studies, we investigated the sequencing depth needed to identify LPS-modulated genes. The pattern for detected pre- and post-LPS DE genes was different from expressed genes. Only a small number of the DE genes were detected at low depths; for the 5 M-read dataset, less than 2% of the DE genes were detected, in contrast to 16% for the expressed genes detected at the same sequencing depth. The detection rate was increased to 45% when the sequencing depth increased to 100 M. Unlike expressed genes, which reached a plateau, for DE genes, the detection rate increased steadily as sequencing depth increased, and in order to detect 80% of the DE genes, 300 M reads were necessary (**Figure 1(A)** **; Table S2**). We note that the curves for pre-LPS and post-LPS samples are overlapping, although the numbers of detected genes were different (**Table S2**). Our results suggest that although expressed genes can be detected at relatively low sequencing depth, the accuracy of the estimated gene expression levels may not be sufficient to determine modest gene expression changes modulated by LPS or in other experimental or disease settings.

#### Correlations of gene expression and differential gene expression across datasets

We next evaluated how accurate the estimated FPKM values and the corresponding fold change of the FPKMs were in all sub-datasets as compared to those obtained from the 500 M-read datasets. For each subset, we calculated the Spearman correlations of the FPKM values with the 500 M-read datasets for both the pre- and post-LPS samples. The spearman correlations were relatively high even for low sequencing depths (**Figure 1(B)**); for the 5 M-read datasets, the correlations with the 500 M-read dataset were 0.88 and 0.74, for the pre- and post-LPS samples, respectively. The correlations were above 0.9 for both pre-LPS and post-LPS samples when the read depths increased to 100 M (**Figure 1(B)** **; Figures S1–S3**). At the same sequencing depth, the correlation for fold-change in gene expression (post-LPS vs. pre-LPS) was always smaller than the corresponding correlations for the FPKM values, and the degree of discrepancy was more pronounced at lower sequencing depth (**Figure 1(B)**). For example, with 10 M reads, the correlations for the pre-LPS and post-LPS FPKM values were 0.88 and 0.75, respectively, whereas the correlation for the fold change of gene expression was only 0.63. This suggests that at lower sequencing depth, one would not only miss a large portion of the DE genes, but would also suffer from a less accurate estimation of the magnitude of gene expression changes.

#### Impact of gene expression levels

Despite overall good correlations between replicates, in one of the first large RNA-Seq studies with technical replicates, Mortazavi et al. [23] observed reduced precision for lower expressed transcripts. In order to assess the impact of gene expression levels on our results, we looked at highly-expressed genes and lowly-expressed genes separately based on their FPKM values in the 500 M-read datasets. “Highly-expressed genes” were defined as those with FPKM values >75th percentile for both the pre-LPS (75^th^ percentile FPKM = 11.46) and post-LPS (75^th^ percentile FPKM = 9.09) samples, and “lowly-expressed genes” were defined as those with the FPKM values <25th percentile (25^th^ percentile FPKM = 1.51 pre-LPS, 0.85 post-LPS).

As expected, the expression level affects how readily a gene was detected. **Figure 1(C)** shows that the detection rate for lowly-expressed genes was significantly lower than that for highly-expressed genes. For highly-expressed genes, 10 M reads could detect 80% (pre-LPS) and 73% (post-LPS) of the genes that were expressed. With the same number of reads, less than 2% of the lowly-expressed genes were detected for both the pre- and post-LPS samples. The gene expression also affected the detection of DE genes. For example, with 100 M reads, among highly-expressed genes, 38% of the DE genes were detected as compared to 20% DE genes detected among lowly-expressed ones (**Figure 1(C)** **; Table S3**). The discrepancy became more pronounced as sequencing depths decreased; with read depths <75 M, none of the DE genes among those lowly-expressed genes were detected, whereas about one third of the DE genes among highly-expressed ones were detected at 75 M. Our results confirmed that read depths have a much larger impact on the detection of expression and especially DE for low abundance genes.

#### Sensitivity and specificity

We also characterized the sensitivity and specificity for DE genes at various sequencing depths. This is an important question because some DE genes detected in datasets with lower sequencing depths were not detected in the 500 M-read datasets, suggesting false positives. Assuming the 500 M-read datasets as the gold standard, we classified DE genes detected in datasets with lower sequencing depths into four categories. A gene was classified as “false positive” (FP) if it was detected in a subset but not in the 500 M datasets; a gene was classified as “false negative” (FN) if it was missed in a subset but detected as DE in the 500 M-read datasets. Similarly, we defined “true positive” (TP) and “true negative” (TN) genes. Based on these definitions, we calculated the numbers of genes in each category, and this allowed us to estimate the sensitivity (i.e., TP/(TP+FN)), specificity (i.e., TN/(FP+TN)), and false discovery rate (FDR) (i.e., FP/(FP+TP)) for DE genes detected at each sequencing depth. As shown in **Figure 1(D)** **(and Table S4)**, overall the specificity was high e.g., 97% even when the sequencing depth was as low as 5 M. However, the sensitivity was strongly dependent on the sequencing depths; with 10 M reads, the sensitivity was only 16%. To achieve 80% sensitivity, 300 M reads were necessary. The FDR was close to 40% with 5 M reads but fell quickly when the sequencing depth increased e.g., to 12% at 100 M reads and to 5% at 300 M reads.

### Impact of Sequencing Depth on Analysis of Alternative Splicing

#### Detection of AS

**Figure 2:**
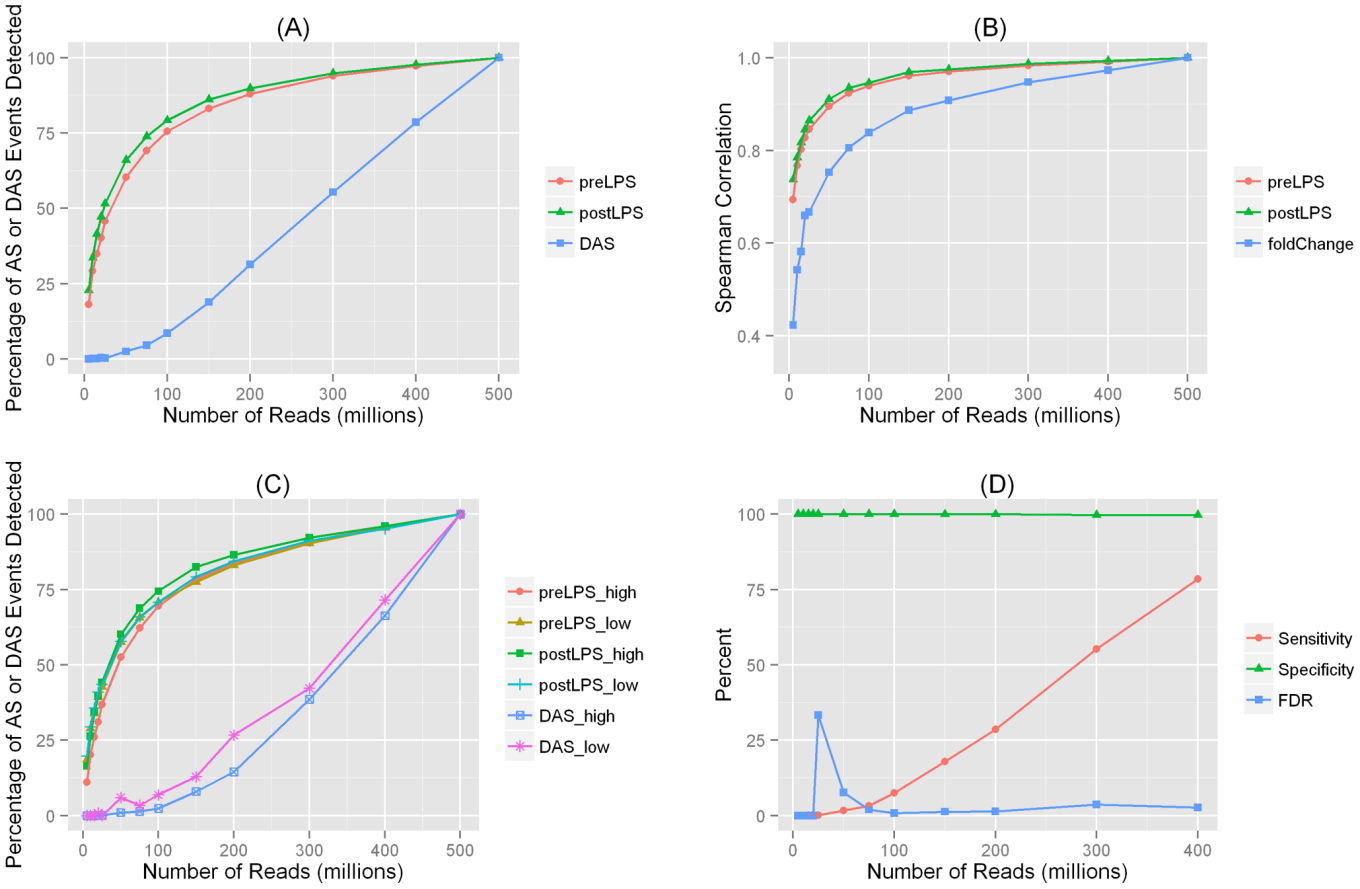
Analysis results for alternative splicing (AS) and differential AS (DAS) in adipose. (**A**) **Percentage of detected alterantive splicing (AS) and differential AS (DAS) events for datasets with various sequencing depths.** PreLPS: detection rate for AS events in the pre-LPS sample; postLPS: detection rate for AS events in the post-LPS sample; DAS: detection rate for DAS events. (**B**) **Spearman correlation between exon or intron inclusion levels in datasets with various sequencing depths and inclusion levels in the 500 M-read datasets.** PreLPS: correlation of inclusion levels in the pre-LPS sample; postLPS: correlation of inclusion levels in the post-LPS sample; fold-change: correlation of the fold change of isoform ratios. (**C**) **Percentage of detected AS and DAS events according to gene expression levels.** preLPS_high: detection rate for AS in highly expressed genes in the pre-LPS sample; preLPS_low: detection rate for AS in lowly expressed genes in the pre-LPS sample; postLPS_high: detection rate for AS in highly expressed genes in the post-LPS sample; postLPS_low: detection rate for AS in lowly expressed genes in the post-LPS sample; DAS_high: detection rate for DAS in highly expressed genes; DAS_low: detection rate for DAS in lowly expressed genes. (**D**) **Performance of DAS events detected in datasets with various sequencing depths.**

An exciting feature of RNA-Seq lies in its ability to study AS, a regulated process during gene expression in which particular exons of a gene may be included or excluded from the final processed mRNAs. In humans, it is estimated that more than 90% of the multiexonic genes are alternatively spliced [5]. An exon was declared to have undergone AS if the exon inclusion level was between zero and one. This type of AS is defined as exon skipping, which is the most common type of AS [5]. Similar inclusion levels were calculated for other types of AS in MATS [20] including mutually exclusive exons, alternative 5′ splice site, alternative 3′ splice site, and intron retention. Since the patterns were consistent across all types of AS events, in our analysis, we considered all AS events together. The patterns of detected AS events were broadly similar to that for expressed genes. AS events could be detected at low sequencing depths e.g., for the pre-LPS sample, 30% of the AS events were detected with 10 M reads and the detection rate quickly reached to 83% when the read depth increased to 150 M (**Figure 2(A)** **; Table S5**).

#### Detection of differential AS

Next, we investigated the sequencing depth needed to study differential AS (DAS) events. Strikingly, none of the DAS events were detected in datasets with sequencing depth below 50 M reads, and only 9% of the DAS events were detected with 100 M reads, in sharp contrast to 76% detection rates for AS events at this depth. In order to detect ∼80% of the DAS events, at least 400 M reads were needed. Since the curve for detection rate did not hit a plateau (**Figure 2(A)**, this suggests that even at higher sequencing depths many DAS events are difficult to identify with current RNA-Seq protocols.

#### Correlations of AS and DAS across datasets

In the analysis of AS, it is important to quantify exon/intron inclusion level as it reflects the relative abundance of different isoforms. For AS and DAS events detected in datasets with various sequencing depths, we calculated the Spearman correlations of the inclusion levels and fold-change in inclusion levels with that in the 500 M-read datasets. As expected, the spearman correlations for inclusion levels were relatively high even at low sequencing depths (**Figure 2(B)** **; Figures S4–S6**); for the 5 M-read datasets, the correlations were 0.69 and 0.74 for the pre- and post-LPS samples, respectively, and quickly reached to 0.9 when the sequencing depths increased to 75 M. At the same sequencing depth, the correlation for fold change of inclusion level was always smaller than the corresponding correlations for the inclusion levels, and further, it was smaller than the fold change for the corresponding gene expression results. For example, with 10 M reads, the correlation for fold change of gene expression was 0.63, whereas the correlation for fold change of inclusion level was only 0.54. This reduced correlation is likely because in datasets with lower sequencing depths, the numbers of junction reads are small which result in more variability in the estimation of inclusion levels.

#### Impact of gene expression levels

The gene expression levels are likely to have an impact on the AS analysis because lowly-expressed genes generally have less junction reads, which are crucial for the analysis of AS. We considered the impact of highly-expressed genes and lowly-expressed genes separately (**Figure 2(C)**). As expected, we detected more AS and DAS events for highly-expressed genes than for lowly-expressed genes (**Table S6**). At the same sequencing depth, about three time more AS events were detected for highly-expressed genes than for lowly-expressed genes. A similar pattern was observed for the detection of DAS. *Sensitivity and* Specificity. Next, we estimated the sensitivity, specificity, FDR, and accuracy for the detected DAS events at various sequencing depths. We calculated the numbers of “false positive”, “false negative”, “true positive”, and “true negative” DAS events by treating results from the 500 M-read datasets as the gold standard. As shown in **Figure 2(D)** **(Table S7)**, the specificity was high across all sequencing depths but the sensitivity was low when sequencing depth was less than 200 M reads. In order to get non-zero sensitivity, 50 M reads were necessary and even with 200 M reads, the sensitivity was only around 30%. Overall in order to achieve 80% sensitivity, at least 400 M reads were needed. The overall FDR was low; however, FDR should be interpreted with caution because the low FDR as well as the noticeable increase at 25 M were driven by the small number of detected DAS events when sequencing depths were low (**Table S7**).

### Impact of Sampling Variations

**Figure 3:**
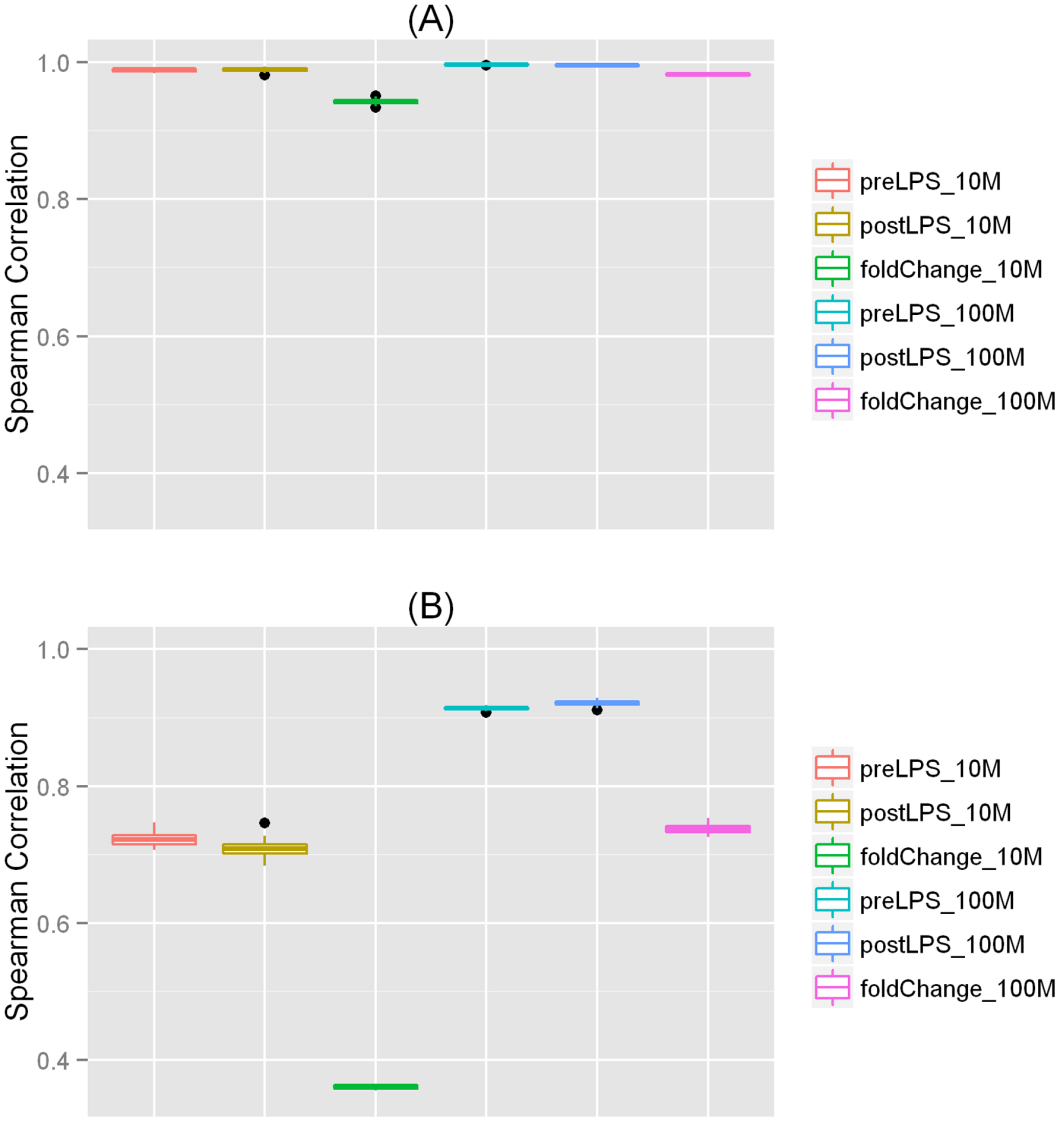
Spearman correlation boxplot for randomly simulated datasets. (**A**) **Boxplot of Spearman correlations for FPKM values and fold change of FPKM values among 10 randomly sampled datasets.** For each sequencing depth (10 M or 100 M), the correlation was calculated for each of the 45 pair-wise comparisons. (**B**) **Boxplot of Spearman correlations for exon/intron inclusion levels and fold change of inclusion levels among 10 randomly sampled datasets.** For each sequencing depth (10 M or 100 M), the correlation was calculated for each of the 45 pair-wise comparisons.

In our primary analysis, we sampled once for each sequencing depth when creating subsets with various numbers of reads. This sampling scheme reflects what happens in real studies because most investigators can only afford to sequence a sample once. However, in experiments with low sequencing depth, it is crucial to evaluate whether the sequenced reads are representative. To evaluate sampling variations, we randomly sampled 100 M reads and 10 M reads from the 500 M-read datasets 10 times for both the pre- and post-LPS samples, and repeated our analyses for gene expression and AS. We observed high correlations for the FPKM values and the fold change of the FPKMs among the 10 samplings (**Figure 3(A)**). As expected, the sampling variation was smaller for the 100 M-read datasets than for the 10 M-read datasets. However, the corresponding correlations for the exon/intron inclusion levels (AS) and the fold change of inclusion levels (DAS) were lower (**Figure 3(B)**), especially when the sequencing depth was as low as 10 M reads. This result suggests that sampling variations has little effect on the analysis of gene expression, but its impact on the analysis of AS is substantial.

Until now, our analyses have been restricted to randomly sampled sequence reads from data generated in a single sequencing run. These selected reads may have less variation than data obtained from different sequencing runs. In order to evaluate this further, we analyzed technical replicate RNA-Seq data generated from the same study subject, where the numbers of reads after filtering were 36 M and 33 M for pre-LPS and post-LPS, respectively (**Table 1**). Based on these data, we examined the numbers of expressed and DE genes, and AS and DAS events and compared these findings to those from data subsets sampled at 36 M and 33 M reads from the 500 M-read datasets. **Table 2** shows that the results are broadly similar, confirming that results obtained from resampled data are representative of empiric data; however, the low overlap in DAS events underscores the lack of sensitivity of low sequence depth for DAS analysis.

**Table 2:** Numbers of expressed genes, differentially expressed (DE) genes, alternative splicing (AS) events and differential AS (DAS) events detected in the technical replicate samples and the resampled data with the same sequencing depth* as the technical replicate.

Sample	Expressed genes(pre-LPS)	Expressed genes(post-LPS)	DE genes	AS events(pre-LPS)	AS events(post-LPS)	DAS events
Technical replicate	15,962	15,324	732	7,506	7,347	12
Resampled data	16,064	15,375	748	8,805	8,586	8
Overlap	15,400	14,756	598	5,562	5,510	1

* 67 M and 65 M reads for the pre-LPS and post-LPS samples, respectively, with 36 M reads and 33 M reads after filtering and removal of reads mapped to mitochondria.

## Discussion

Tissue and cell-specific transcriptomic modulation in disease and during experimental interventions are an emerging interest in the study of cardiometabolic diseases [24]. In this study, using deeply sequenced RNA-Seq data derived from adipose of a healthy individual before and after systemic administration of LPS, we investigated the sequencing depths needed for studies of various types of transcriptomic variations. In particular, we examined what sequencing depths were needed for studying gene expression and AS. We found that to detect expressed genes, ∼100 M reads (after filtering and removal of mitochondrial reads) were needed. However, in order to detect DE genes, the requirement on sequencing depth was much higher. In general, ∼300 M reads were needed to detect 80% of the identified DE genes. We also investigated the sequencing depth needed for the analysis of AS. To detect AS events, ∼150 M reads were necessary; however, to detect DAS events, a much higher sequencing depth was needed; at least 400 M reads were necessary to achieve an 80% detection rate. These findings provide a practical guide and cautionary note for researchers when designing RNA-Seq experiments in cells and tissues of direct relevance to cardiometabolic disease.

The sequencing depth needed for a given study depends on several factors including genome size, transcriptome complexity and objectives of the study. Depending on the purpose of the analysis, the requirement of sequencing depth varies. In most transcriptomics studies, quantifying gene expression is the major objective. As shown by several groups [10], [11], [12] and confirmed by us, there is a certain sequencing depth that is sufficient for detection of expressed genes, implying that increasing sequencing depth after reaching a certain threshold has little impact on gene detection. For example, our data suggest that after the sequencing depth reached 150 M filtered reads, the percentage of additionally detected genes became less pronounced. This indicates that ∼300 M raw sequence reads were needed, equivalent to 1.5 lanes per sample if sequencing is performed using Illumina’s HiSeq 2000. However, our analysis demonstrates for the first time that reliable detection of DE genes, at least in adipose, requires much deeper sequencing than has been applied typically. Further, by separating genes by expression levels, we observed substantial difference between highly-expressed genes and lowly-expressed genes in terms of the detection of expressed genes and DE genes. Detection of low abundance genes and of DE of such genes was greatly impacted by sequencing depth. This finding is particularly relevant to study design when the goal is to detect DE genes that are novel and of low expression.

A unique strength of RNA-Seq is its capacity to identify AS and DAS in an unbiased manner. Detection of AS in our adipose RNA-Seq data had a similar pattern to that for gene expression except that greater sequencing depth was required to detect AS events; for example, to achieve 80% detection rate, 100 M filtered reads were needed for gene expression but 150 M filtered reads were necessary for AS. In a manner that parallels that for the impact of gene expression level on the analysis of gene expression, low number of junction reads (i.e., low expression) greatly impacted the detection of AS and required much higher sequencing depth.

Detection of tissue or cell-specific disease or drug-related DAS may provide novel insight into specific genes and proteins that modulate cardiometabolic disease and therapeutic responses. Therefore, it is of great interest to identify DAS under different disease and experimental conditions. Strikingly, the curves for DAS detection rate did not reach a plateau suggesting that increasing sequencing depth even beyond 500 M filtered reads would provide more reliable detection of DAS in our adipose data. Since RNA-Seq remains expensive and most investigations cannot afford to sequence even to 500 M reads, results reported from most experiments with lower sequencing depth likely represent a very incomplete picture for DAS. For example, our data suggest that a sequencing depth of 200 M reads would detect only ∼30% of DAS and even less for those AS events with relatively low numbers of junctions reads.

The coverage we report here represents the number of reads after filtering of inappropriate alignment and removal of reads mapped to the mitochondrial genome. This may vary by alignment programs and filtering criteria, and thus may vary from study to study. Further, our reported coverage represents a lower bound of the required depth since we were stringent in our filtering. As better algorithms are developed to improve read mapping, we anticipate that more reads will be used in gene expression quantification and AS analysis, and thus reduce the numbers of reads needed to obtain robust analysis results.

Our study has several unique strengths but also limitations. To our knowledge, this is the first analysis of sequencing depth on transcriptomic profiling of differential gene expression and DAS in human adipose. We applied very deep RNA-Seq, paired tissue sampling, resampling of sequence reads, and empirical technical replication of RNA-Seq data in our approach. Our analysis was restricted, however, to a single individual and it is possible that findings could vary across individuals and with increasing numbers of individuals studied. Although experimental endotoxemia is not a disease model or study design typically used in the study of cardiometabolic disease, several lines of evidence suggest that controlled activation of innate immunity in healthy humans may be informative in cardiometabolic disease [14], [15]. We and others have shown that experimental endotoxemia induces insulin resistance [14] and atherogenic lipoprotein changes [25] while observational studies show that sepsis and chronic infection [26], [27] induce metabolic derangements resembling those observed in obesity, type 2 diabetes and atherosclerosis. Furthermore, LPS activation of TLR-4 signaling has a well established and robust impact on regulation of gene expression and AS, thus providing a highly informative model for our analysis of the sequencing depth required to detect LPS-modulated DE and DAS in humans. While we acknowledge that our focus was restricted to adipose tissue and that findings could vary across distinct cells and tissues, we note that analysis of adipose tissue transcripts has established its utility in informing our understanding of complex cardiometabolic disorders [28], [29], [30]. Finally, we acknowledge that our findings might be sensitive to methods used in alignment, filtering, and analyses as well as the assumption that the 500 M-read datasets represent a gold standard in our analyses.

Our analysis was restricted to a single individual. Although not typical (i.e., without biological replicates), our results are particularly relevant to the design of the GENE study and studies that involve paired samples (e.g., pre vs. post treatment). In the GENE study, each participant was administrated low-dose LPS, and we are interested in identifying transcriptomic variations induced by LPS in each individual. This is important as our clinical investigations revealed substantial phenotypic variations among individuals despite their similar baseline characteristics LPS [17]. The typical differential expression/splicing analyses with biological replicates will miss signals that are present only in a small number of individuals. We note that the analysis of single individuals is also relevant to cancer transcriptomics studies when comparing paired tumor and normal tissues in which the analysis is typically done at the individual level.

To assess the generalizability of our findings in other tissues, we also analyzed the deeply sequenced RNA-Seq data obtained from blood of the same individual. We analyzed the blood RNA-Seq data using the same pipeline as employed for adipose. The patterns for gene expression and AS results are broadly similar to adipose (**Figures S7 and S8**), suggesting that our conclusions on required sequencing depths might be generalized to other tissue types.

In summary, recent development in sequencing technologies has allowed us to obtain deep coverage of the human transcriptome at single-base resolution. We report the first study that evaluates the appropriate sequencing depth for studying differential gene expression and differential AS in human adipose using RNA-Seq. Our results show that a much higher sequencing depth is needed to reliably identify DAS events and even DE genes compared to that needed to detect gene expression or AS. While contemporary sequencing depths in RNA-Seq studies of human diseases may provide novel and important findings, it is likely that most lack coverage to reliably detect the full spectrum of disease relevant differential gene expression and AS. The knowledge generated from this study provides a realistic foundation for applications of RNA-Seq in the study of tissue and cell-specific transcriptomic modulation within cardiometabolic disorders.

### Accession Numbers

RNA-seq data have been deposited in the Gene Expression Omnibus (GEO) database (accession number GSE46323).

## Supporting Information

Figure S1 **FPKM values estimated from datasets with various sequencing depths for the pre-LPS sample.** Shown are the values of –log10(FPKM +1). X-axis is for the 500 M-read dataset and Y-axis is for datasets of lower sequencing depths.
(TIF)

Figure S2 **FPKM values estimated from datasets with various sequencing depths for the post-LPS sample.** Shown are the values of –log10(FPKM +1). X-axis is for the 500 M-read dataset and Y-axis is for datasets of lower sequencing depths.
(TIF)

Figure S3 **Fold change of FPKM values estimated from datasets with various sequencing depths.** Shown are the values of –log10(fold change +0.01). X-axis is for the 500 M-read dataset and Y-axis is for datasets of lower sequencing depths.
(TIF)

Figure S4 **Exon/intron inclusion levels estimated from datasets with various sequencing depths for the pre-LPS sample.** X-axis is for the 500 M-read dataset and Y-axis is for datasets of lower sequencing depths.
(TIF)

Figure S5 **Exon/intron inclusion levels estimated from datasets with various sequencing depths for the post-LPS sample.** X-axis is for the 500 M-read dataset and Y-axis is for datasets of lower sequencing depths.
(TIF)

Figure S6 **Fold change of inclusion levels estimated from datasets with various sequencing depths.** Shown are the values of –log10(fold change +0.01). X-axis is for the 500 M-read dataset and Y-axis is for datasets of lower sequencing depths.
(TIF)

Figure S7 **Analysis results for differentially expressed (DE) genes in blood (A) Percentage of detected expressed genes and differentially expressed (DE) genes for datasets with various sequencing depths in blood.** PreLPS: detection rate for expressed genes in the pre-LPS sample; post-PLS: detection rate for expressed genes in the post-LPS sample; DE: detection rate for DE genes. The curves for pre-LPS and post-LPS samples overlap, although the numbers of detected genes were different (Table S1). **(B) Spearman correlation between FPKM values in datasets with various sequencing depths and FPKM values in the 500 M-read datasets in blood.** PreLPS: correlation of FPKM values in the pre-LPS sample; postLPS: correlation of FPKM values in the post-LPS sample; fold-change: correlation of the fold change of FPKM values. **(C) Percentage of detected DE genes according to gene expression levels in blood.** PreLPS_high: detection rate for gene expression in highly expressed genes in the pre-LPS sample; preLPS_low: detection rate for gene expression in lowly expressed genes in the pre-LPS sample; postLPS_high: detection rate for gene expression in highly expressed genes in the post-LPS sample; postLPS_low: detection rate for gene expression in lowly expressed genes in the post-LPS sample; DE_high: detection rate for DE genes in highly expressed genes; DE_low: detection rate for DE genes in lowly expressed genes. **(D) Performance of DE genes detected in datasets with various sequencing depths in blood.**
(TIF)

Figure S8 **Analysis results for alternative splicing (AS) and differential AS (DAS) in blood. (A) Percentage of detected alterantive splicing (AS) and differential AS (DAS) events for datasets with various sequencing depths in blood.** PreLPS: detection rate for AS events in the pre-LPS sample; postLPS: detection rate for AS events in the post-LPS sample; DAS: detection rate for DAS events. **(B) Spearman correlation between exon or intron inclusion levels in datasets with various sequencing depths and inclusion levels in the 500 M-read datasets in blood.** PreLPS: correlation of inclusion levels in the pre-LPS sample; postLPS: correlation of inclusion levels in the post-LPS sample; fold-change: correlation of the fold change of isoform ratios. **(C) Percentage of detected AS and DAS events according to gene expression levels in blood.** preLPS_high: detection rate for AS in highly expressed genes in the pre-LPS sample; preLPS_low: detection rate for AS in lowly expressed genes in the pre-LPS sample; postLPS_high: detection rate for AS in highly expressed genes in the post-LPS sample; postLPS_low: detection rate for AS in lowly expressed genes in the post-LPS sample; DAS_high: detection rate for DAS in highly expressed genes; DAS_low: detection rate for DAS in lowly expressed genes. **(D) Performance of DAS events detected in datasets with various sequencing depths in blood.**
(TIF)

Table S1 **Characteristics of GENE European ancestry participants at (A) baseline and (B) during endotoxemia.**
(DOCX)

Table S2 **The numbers and percentages of detected expressed genes and DE genes in datasets with various sequencing depths.**
(XLSX)

Table S3 **The numbers and percentages of detected expressed genes and DE genes at each sequencing depth for genes defined as "highly expressed" or "lowly expressed".**
(XLSX)

Table S4 **Sensitivity, specificity, and FDR for detected DE genes in datasets of various sequencing depths.**
(XLSX)

Table S5 **The numbers and percentages of detected AS and DAS events in datasets with various sequencing depths.**
(XLSX)

Table S6 **The numbers and percentages of detected AS and DAS events at each sequencing depth for genes defined as "highly expressed" or "lowly expressed".**
(XLSX)

Table S7 **Sensitivity, specificity, and FDR for detected DAS events in datasets of various sequencing depths.**
(XLSX)

Methods S1 **Supplementary methods.**
(DOC)
