# Supplementary figures and images for: Evaluating the Impact of Sequencing Depth on Transcriptome Profiling in Human Adipose
Source: PLoS One. 2013 Jun 24;8(6):e66883. doi: 10.1371/journal.pone.0066883 (PMC3691247; doi:10.1371/journal.pone.0066883)

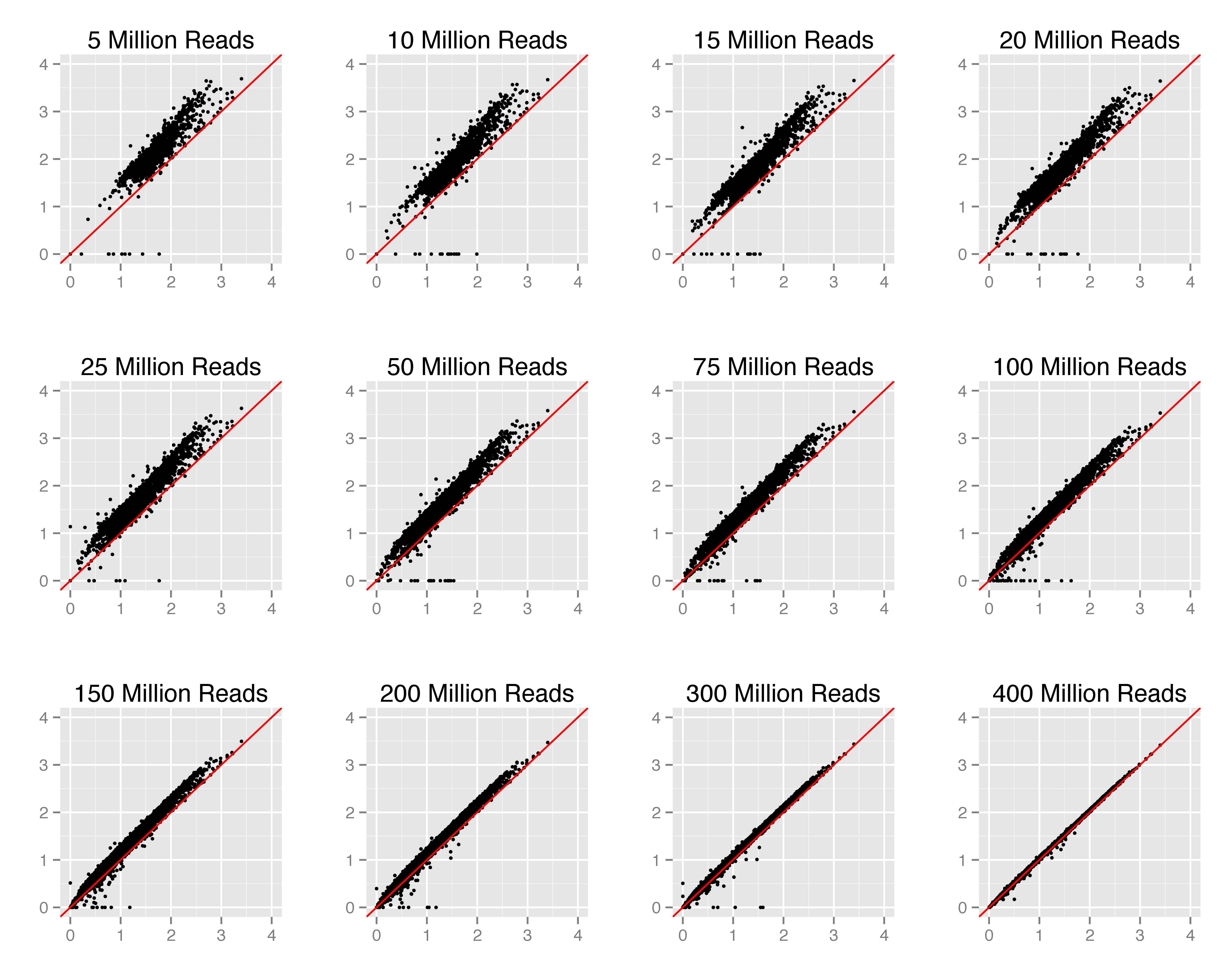

Supplement: Figure S1 — FPKM values estimated from datasets with various sequencing depths for the pre-LPS sample. Shown are the values of –log10(FPKM +1). X-axis is for the 500 M-read dataset and Y-axis is for datasets of lower sequencing depths. (TIF) [file pone.0066883.s001.tif]

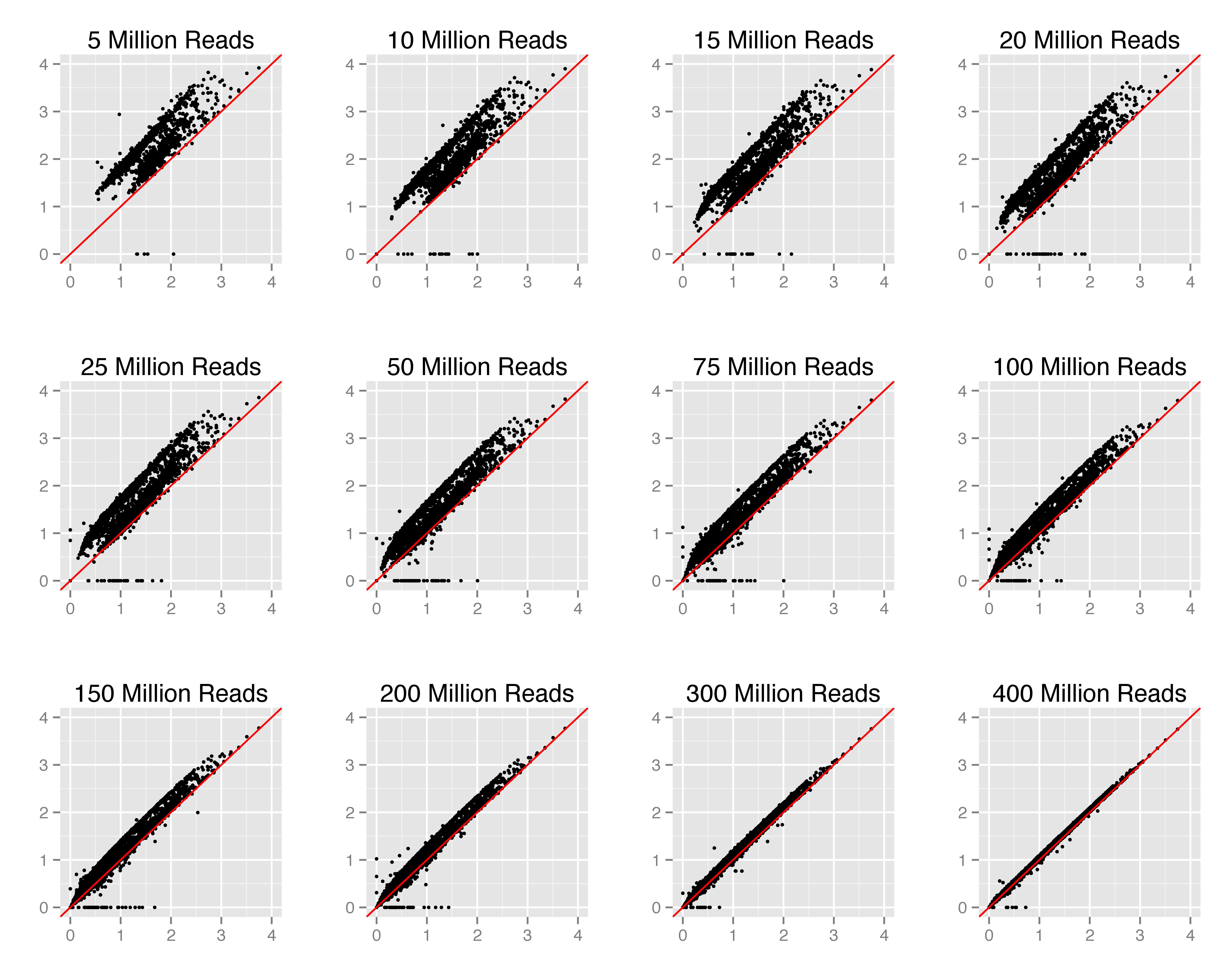

Supplement: Figure S2 — FPKM values estimated from datasets with various sequencing depths for the post-LPS sample. Shown are the values of –log10(FPKM +1). X-axis is for the 500 M-read dataset and Y-axis is for datasets of lower sequencing depths. (TIF) [file pone.0066883.s002.tif]

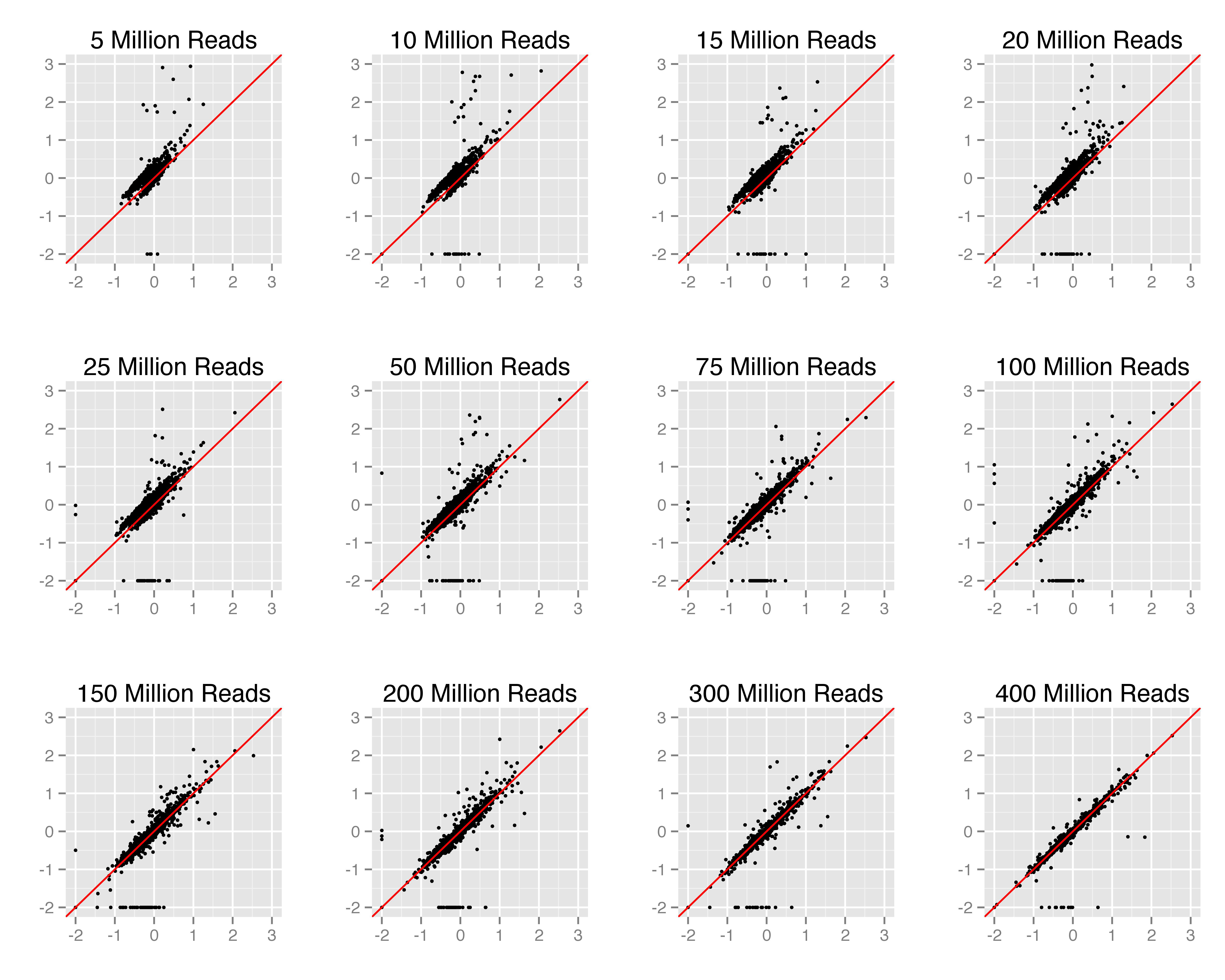

Supplement: Figure S3 — Fold change of FPKM values estimated from datasets with various sequencing depths. Shown are the values of –log10(fold change +0.01). X-axis is for the 500 M-read dataset and Y-axis is for datasets of lower sequencing depths. (TIF) [file pone.0066883.s003.tif]

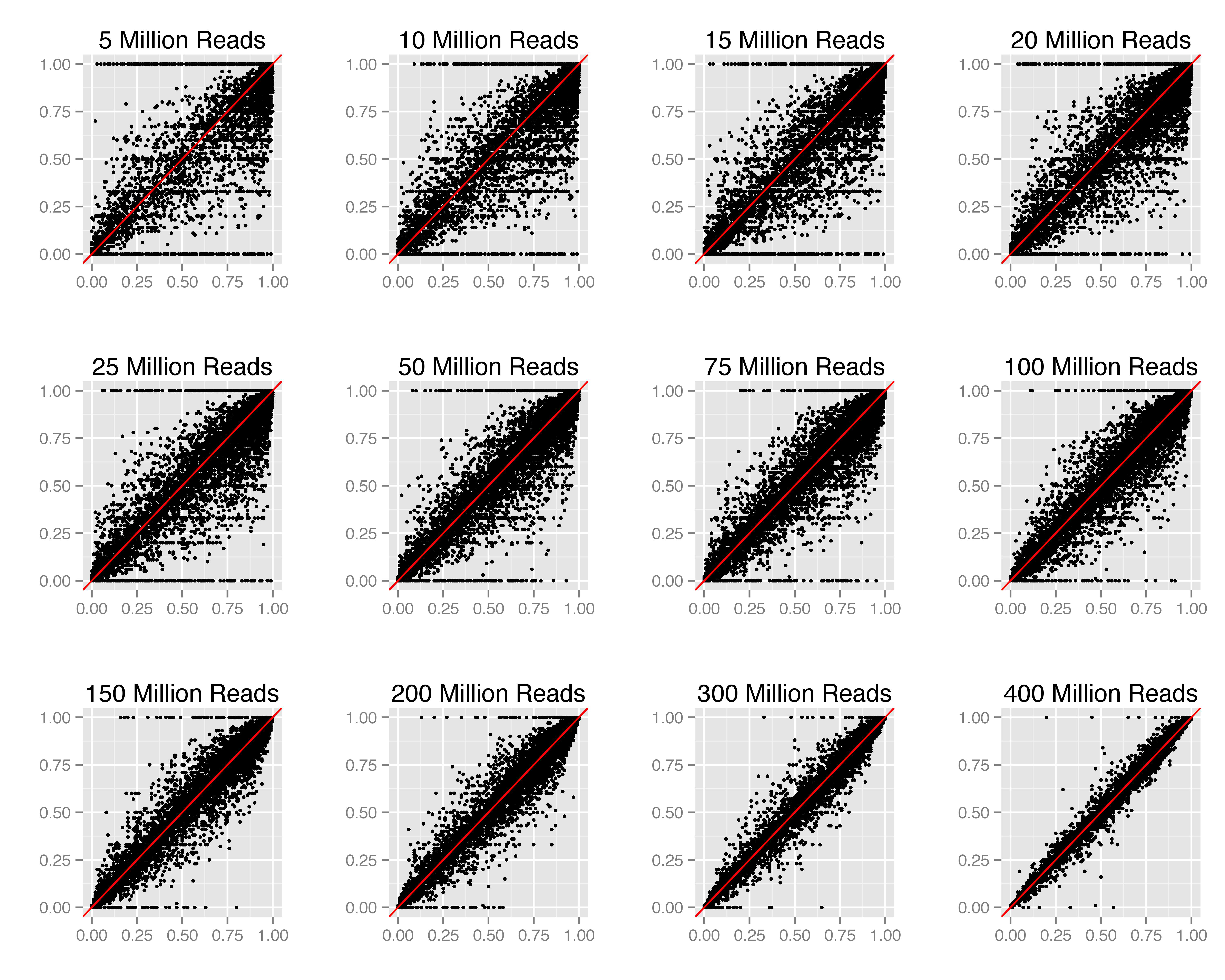

Supplement: Figure S4 — Exon/intron inclusion levels estimated from datasets with various sequencing depths for the pre-LPS sample. X-axis is for the 500 M-read dataset and Y-axis is for datasets of lower sequencing depths. (TIF) [file pone.0066883.s004.tif]

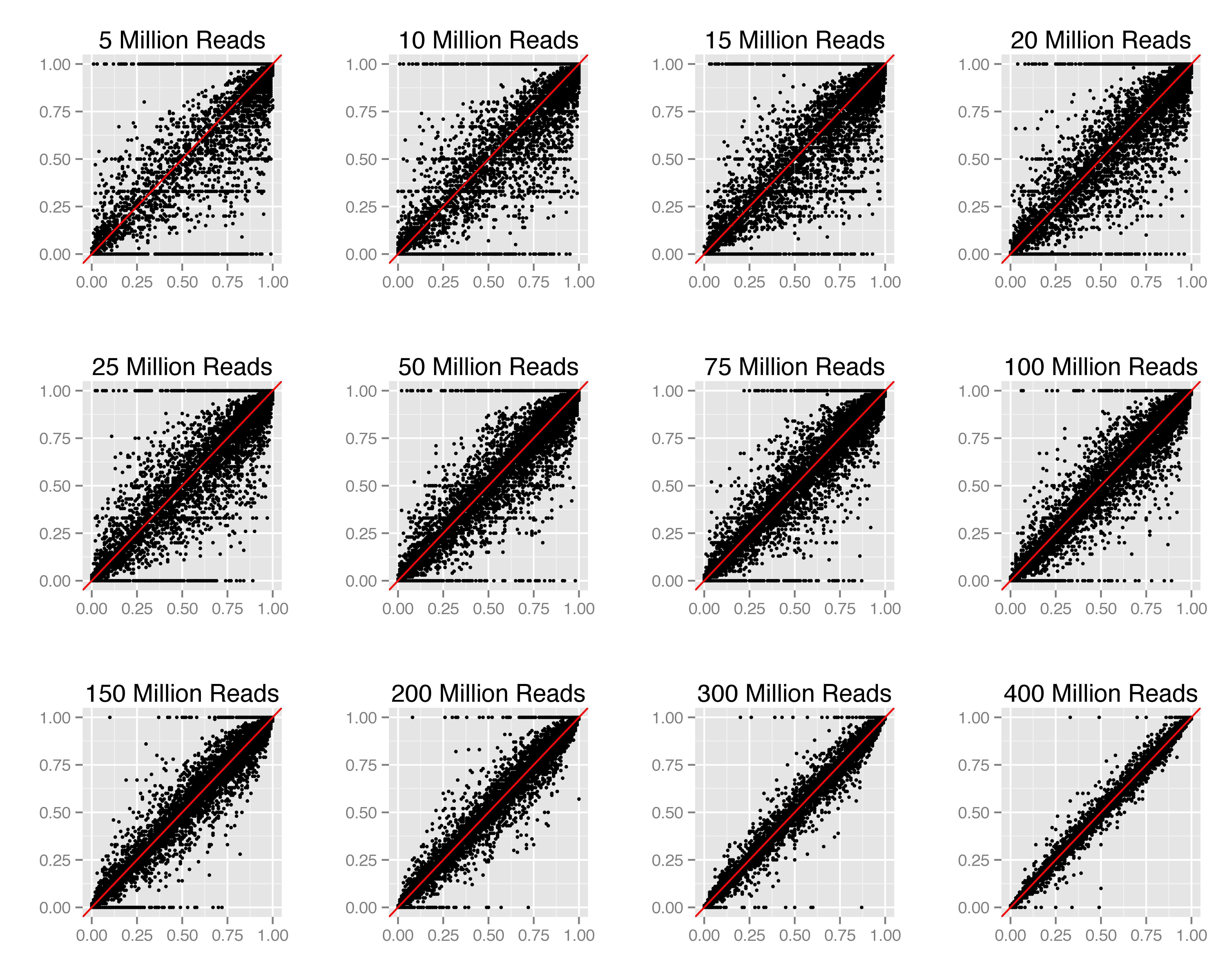

Supplement: Figure S5 — Exon/intron inclusion levels estimated from datasets with various sequencing depths for the post-LPS sample. X-axis is for the 500 M-read dataset and Y-axis is for datasets of lower sequencing depths. (TIF) [file pone.0066883.s005.tif]

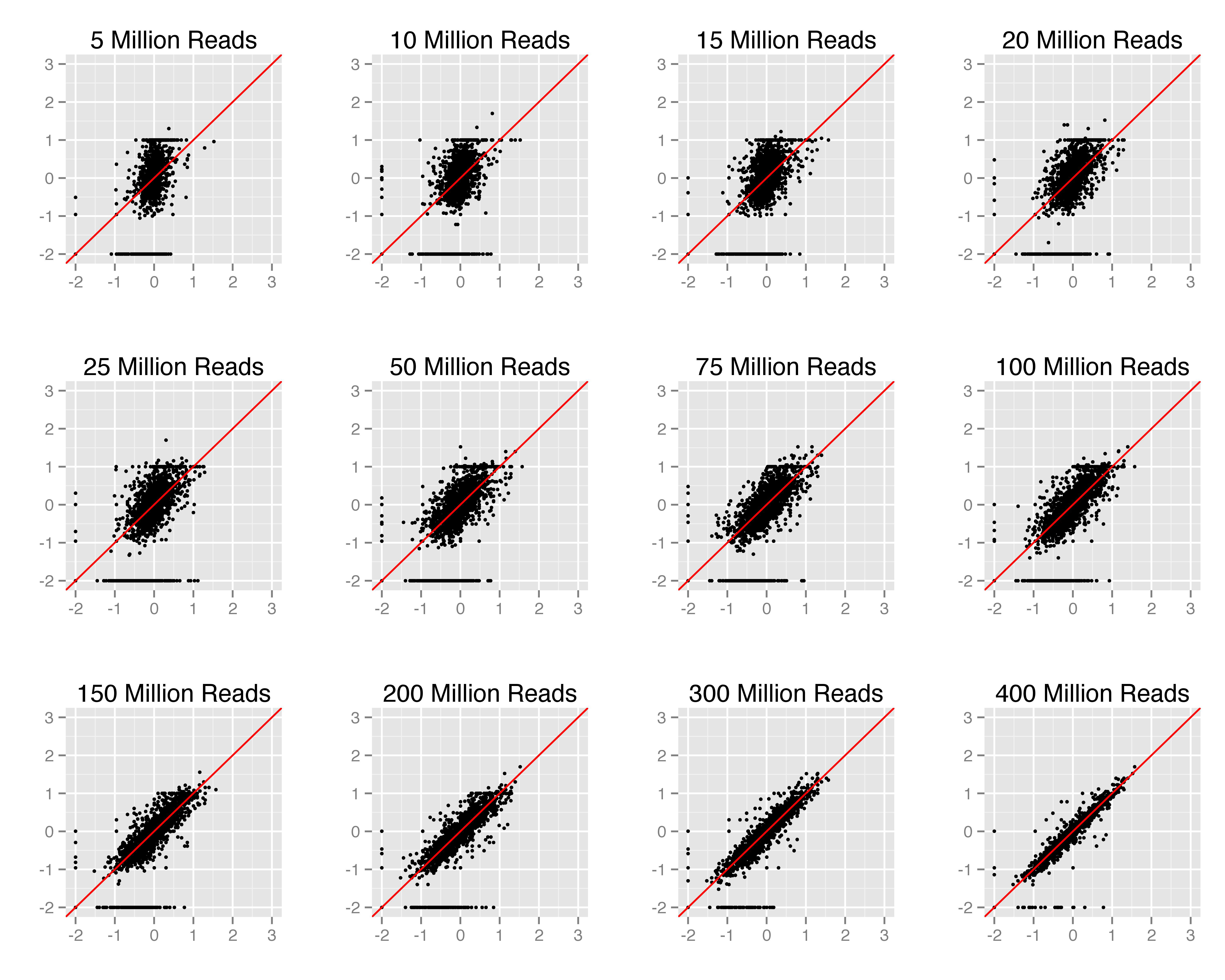

Supplement: Figure S6 — Fold change of inclusion levels estimated from datasets with various sequencing depths. Shown are the values of –log10(fold change +0.01). X-axis is for the 500 M-read dataset and Y-axis is for datasets of lower sequencing depths. (TIF) [file pone.0066883.s006.tif]

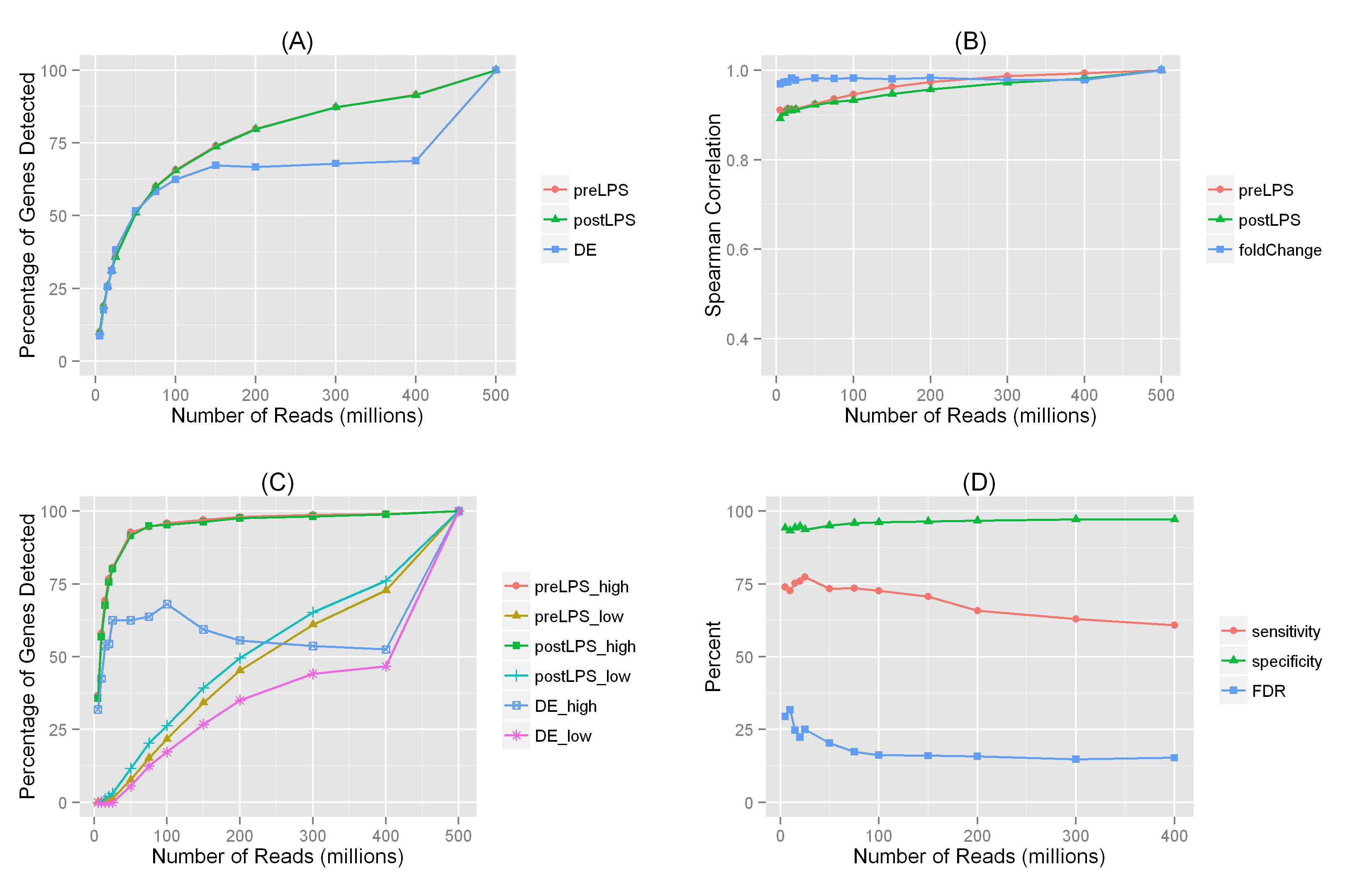

Supplement: Figure S7 — Analysis results for differentially expressed (DE) genes in blood (A) Percentage of detected expressed genes and differentially expressed (DE) genes for datasets with various sequencing depths in blood. PreLPS: detection rate for expressed genes in the pre-LPS sample; post-PLS: detection rate for expressed genes in the post-LPS sample; DE: detection rate for DE genes. The curves for pre-LPS and post-LPS samples overlap, although the numbers of detected genes were different (Table S1). (B) Spearman correlation between FPKM values in datasets with various sequencing depths and FPKM values in the 500 M-read datasets in blood. PreLPS: correlation of FPKM values in the pre-LPS sample; postLPS: correlation of FPKM values in the post-LPS sample; fold-change: correlation of the fold change of FPKM values. (C) Percentage of detected DE genes according to gene expression levels in blood. PreLPS_high: detection rate for gene expression in highly expressed genes in the pre-LPS sample; preLPS_low: detection rate for gene expression in lowly expressed genes in the pre-LPS sample; postLPS_high: detection rate for gene expression in highly expressed genes in the post-LPS sample; postLPS_low: detection rate for gene expression in lowly expressed genes in the post-LPS sample; DE_high: detection rate for DE genes in highly expressed genes; DE_low: detection rate for DE genes in lowly expressed genes. (D) Performance of DE genes detected in datasets with various sequencing depths in blood. (TIF) [file pone.0066883.s007.tif]

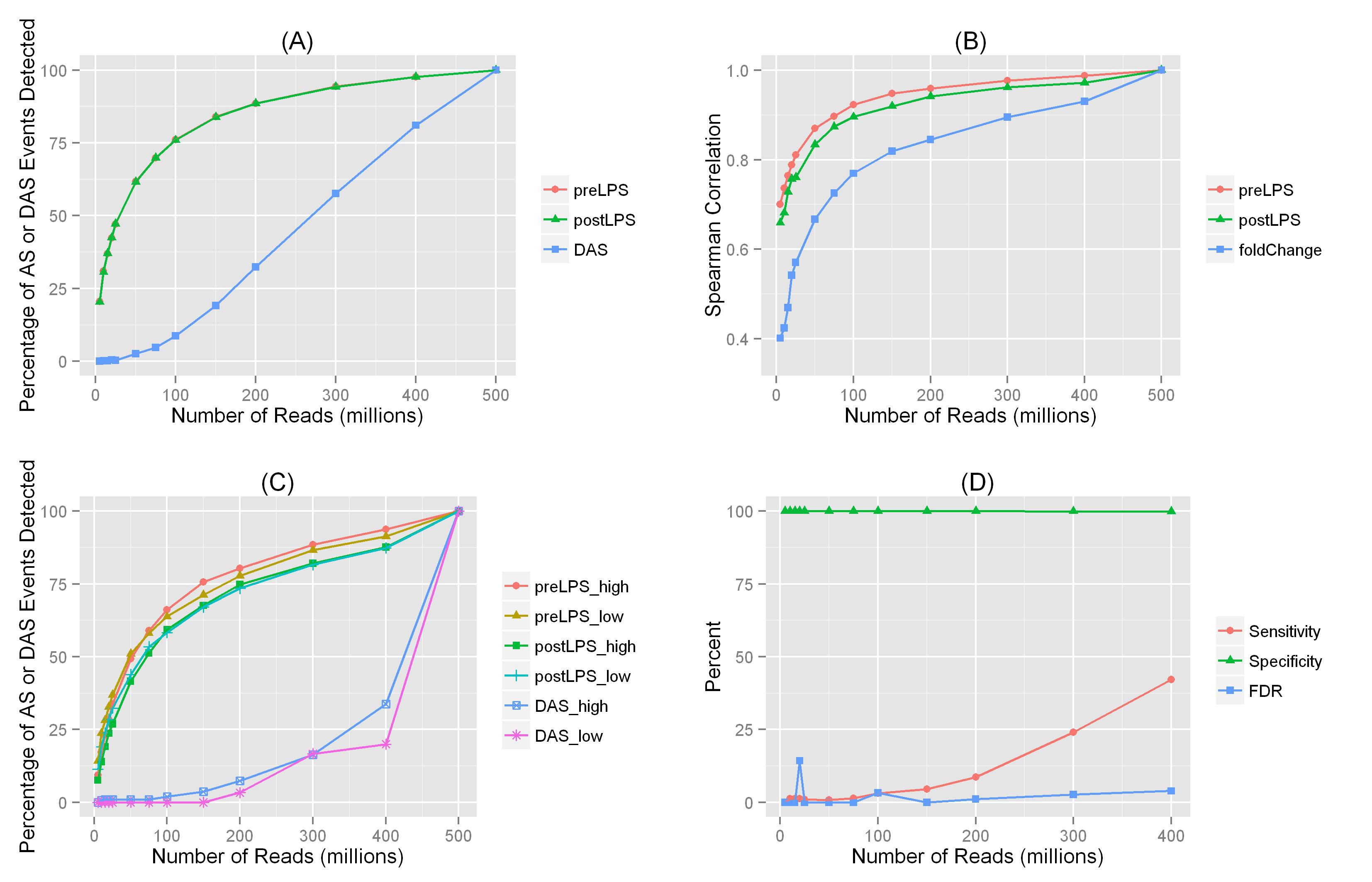

Supplement: Figure S8 — Analysis results for alternative splicing (AS) and differential AS (DAS) in blood. (A) Percentage of detected alterantive splicing (AS) and differential AS (DAS) events for datasets with various sequencing depths in blood. PreLPS: detection rate for AS events in the pre-LPS sample; postLPS: detection rate for AS events in the post-LPS sample; DAS: detection rate for DAS events. (B) Spearman correlation between exon or intron inclusion levels in datasets with various sequencing depths and inclusion levels in the 500 M-read datasets in blood. PreLPS: correlation of inclusion levels in the pre-LPS sample; postLPS: correlation of inclusion levels in the post-LPS sample; fold-change: correlation of the fold change of isoform ratios. (C) Percentage of detected AS and DAS events according to gene expression levels in blood. preLPS_high: detection rate for AS in highly expressed genes in the pre-LPS sample; preLPS_low: detection rate for AS in lowly expressed genes in the pre-LPS sample; postLPS_high: detection rate for AS in highly expressed genes in the post-LPS sample; postLPS_low: detection rate for AS in lowly expressed genes in the post-LPS sample; DAS_high: detection rate for DAS in highly expressed genes; DAS_low: detection rate for DAS in lowly expressed genes. (D) Performance of DAS events detected in datasets with various sequencing depths in blood. (TIF) [file pone.0066883.s008.tif]
