# Supplementary material for: Evaluating the Impact of Sequencing Depth on Transcriptome Profiling in Human Adipose
Source: PLoS One. 2013 Jun 24;8(6):e66883. doi: 10.1371/journal.pone.0066883 (PMC3691247; doi:10.1371/journal.pone.0066883)
Supplement: Table S1 — Characteristics of GENE European ancestry participants at (A) baseline and (B) during endotoxemia. (DOCX) [file pone.0066883.s009.docx]

**Supplementary Table 1:** Characteristics of GENE European ancestry participants at (A) baseline and (B) during endotoxemia.

|  | **Male N=99** | | **Female N=93** | | **Study Subject** |
| --- | --- | --- | --- | --- | --- |
|  | **Mean** | **SD** | **Mean** | **SD** | **Female** |
| **Age (Years)** | 25.6 | 6.7 | 23.9 | 4.0 | 34 |
| **Total Cholesterol (mg/dL)** | 150.9 | 33.2 | 149.5 | 25.8 | 147 |
| **HDL-Cholesterol (mg/dL)** | 47.1 | 10.3 | 54.8 | 13.2 | 66 |
| **Triglycerides (mg/dL)** | 83.9 | 44.9 | 77.8 | 33.1 | 63 |
| **LDL-Cholesterol (mg/dL)** | 87.0 | 30.3 | 79.2 | 24.3 | 68 |
| **Systolic BP (mmHg)** | 112.8 | 10.8 | 108.7 | 10.0 | 112 |
| **Diastolic BP (mmHg)** | 65.0 | 8.8 | 62.0 | 7.8 | 66 |
| **Glucose (mg/dL)** | 87.1 | 13.3 | 82.0 | 15.5 | 79 |

**(A)**

**(B)**

|  | **Male N=99** | | **Female N=93** | | **Study Subject** |
| --- | --- | --- | --- | --- | --- |
|  | **Mean** | **SD** | **Mean** | **SD** | **Female** |
| **IL-6 (pg/ml) Baseline** | 2.5 | 1.5 | 3.2 | 1.7 | 3.9 |
| **IL-6 (pg/ml) Peak (2hour)** | 151.7 | 142.6 | 198.3 | 181.7 | 293.5 |
| **Fold Change IL-6** | 60 |  | 62 |  | 76 |
| **Percentile rank Peak IL6 (2 hour)** |  |  |  |  | 84.3 |
| **TNFα pg/ml Baseline** | 1.3 | 0.8 | 1.3 | 0.8 | 0.8 |
| **TNFα pg/ml Peak (2 hour)** | 50.1 | 35.3 | 57.8 | 51.7 | 50.1 |
| **Fold Change TNFa** | 38 |  | 44 |  | 65 |
| **Percentile rank Peak TNFα** |  |  |  |  | 63.5 |
| **CRP mg/L Baseline** | 1.1 | 3.6 | 1.5 | 2.6 | 0.8 |
| **CRP mg/L Peak (24 hour)** | 18.4 | 8.7 | 16.4 | 8.1 | 19.7 |
| **Percentile rank Peak CRP** |  |  |  |  | 67.5 |
| **SAA (mg/L) Baseline** | 7.1 | 32.1 | 7.3 | 30.9 | 2.6 |
| **SAA (mg/L) Peak (24 hour)** | 95.1 | 42.5 | 81.7 | 46.1 | 81.4 |
| **Percentile rank Peak SAA** |  |  |  |  | 51.7 |

HDL: high density lipoprotein; LDL: low density lipoprotein; IL-6: interleukin-6; TNFα: tumor necrosis factor alpha; CRP: C reactive protein; SAA: serum amyloid A. SD: standard deviation.
