# Supplementary material for: Evaluating the Impact of Sequencing Depth on Transcriptome Profiling in Human Adipose
Source: PLoS One. 2013 Jun 24;8(6):e66883. doi: 10.1371/journal.pone.0066883 (PMC3691247; doi:10.1371/journal.pone.0066883)
Supplement: Methods S1 — Supplementary methods. (DOC) [file pone.0066883.s016.doc]

Supplementary Methods

Poly-A library preparation and sequencing were performed at the Penn Genome Frontiers Institute’s High-Throughput Sequencing Facility per standard protocols. Briefly, we generated first-strand cDNA using random hexamer-primed reverse transcription, followed by secondstrand cDNA synthesis using RNase H and DNA polymerase, and ligation of sequencing adapters using the TruSeq RNA Sample Preparation Kit (Illumina, San Diego, CA). Fragments of ~350 bp were selected by gel electrophoresis, followed by 15 cycles of PCR amplification. The prepared libraries were then sequenced using Illumina’s HiSeq 2000 with four lanes per sample which generated 2×101 bp paired-end reads. Technical replicate RNA-Seq data from the same individual were generated from independent library preparations and sequenced using two samples per lane.
